# Deciphering the photochemical mechanisms describing the UV-induced processes occurring in solvated guanine monophosphate

**DOI:** 10.3389/fchem.2015.00029

**Published:** 2015-04-20

**Authors:** Salvatore F. Altavilla, Javier Segarra-Martí, Artur Nenov, Irene Conti, Ivan Rivalta, Marco Garavelli

**Affiliations:** ^1^Dipartimento di Chimica “G. Ciamician,” Università di BolognaBologna, Italy; ^2^École Normale Supérieure de Lyon, Centre National de la Recherche Scientifique, UMR 5182, Université de LyonLyon, France

**Keywords:** DNA, purine, CASSCF/CASPT2, photochemistry, QM/MM, photostability, GMP, guanine

## Abstract

The photophysics and photochemistry of water-solvated guanine monophosphate (GMP) are here characterized by means of a multireference quantum-chemical/molecular mechanics theoretical approach (CASPT2//CASSCF/AMBER) in order to elucidate the main photo-processes occurring upon UV-light irradiation. The effect of the solvent and of the phosphate group on the energetics and structural features of this system are evaluated for the first time employing high-level *ab initio* methods and thoroughly compared to those *in vacuo* previously reported in the literature and to the experimental evidence to assess to which extent they influence the photoinduced mechanisms. Solvated electronic excitation energies of solvated GMP at the Franck-Condon (FC) region show a red shift for the ππ^*^ L_a_ and L_b_ states, whereas the energy of the oxygen lone-pair nπ^*^ state is blue-shifted. The main photoinduced decay route is promoted through a ring-puckering motion along the bright lowest-lying L_a_ state toward a conical intersection (CI) with the ground state, involving a very shallow stationary point along the minimum energy pathway in contrast to the barrierless profile found in gas-phase, the point being placed at the end of the minimum energy path (MEP) thus endorsing its ultrafast deactivation in accordance with time-resolved transient and photoelectron spectroscopy experiments. The role of the nπ^*^ state in the solvated system is severely diminished as the crossings with the initially populated L_a_ state and also with the L_b_ state are placed too high energetically to partake prominently in the deactivation photo-process. The proposed mechanism present in solvated and *in vacuo* DNA/RNA chromophores validates the intrinsic photostability mechanism through CI-mediated non-radiative processes accompanying the bright excited-state population toward the ground state and subsequent relaxation back to the FC region.

## Introduction

The genomic material and the mechanisms that process the disposal of the excess energy attained upon UV-light irradiation are of paramount importance from both biomedical and biotechnological standpoints. The initially populated excited states and their fate along the distinct deactivation routes present in the DNA/RNA double helix chains relate simultaneously to the intrinsic photostability of the genomic material (Callis, 1983; Crespo-Hernandez et al., 2004; Kohler, 2010), as well as to the damaging photo-reactions that ultimately yield mutations and single- and double-strand breaks that have been associated to increasingly featured diseases like skin cancer (Cadet et al., 2012; Noonan et al., 2012; Giussani et al., 2013b; Brash, 2015). Besides the biological relevance, the intricate photophysical properties of nucleobases can be also exploited to design optical photo-responsive nanomachines among a long list of prospective applications (Kamiya and Asanuma, 2014), yet it is their biological relevance that focuses our attention.

From a bio-physical and chemical point of view, fundamental knowledge on the deactivation processes, including the associated underlying molecular motions, are essential to rationalize the intrinsic photostability of the genomic material and to characterize the aforementioned routes leading to deleterious photochemical reactions (Markovitsi et al., 2010; Cadet et al., 2012). These different pathways increase in number and complexity as the size of the DNA/RNA single- or double-stranded chain enlarges, as the different de-excitation routes interact with one another yielding a ramification of complex photoinduced pathways that become extremely hard to discern, disentangle, and comprehend. This increasing degree of complexity arises partially due to the intermolecular interactions occurring from inter- and intra-strand conformations between the nucleobases compressing the DNA/RNA chains, related to their hydrogen-bonding motifs and π-stacking interactions, respectively. Extensive experimental work has been carried out recently in order to identify and separate the different contributions arising from these interactions, providing indicative fingerprints in the appearance of a long-lived spectroscopic signal originating from a π-stacking conformation within intra-strand sequences (Takaya et al., 2008; Su et al., 2012; Vayá et al., 2012; Chen et al., 2013, 2014; Chen and Kohler, 2014; Plasser et al., 2015) and to an ultrafast proton/hydrogen transfer stemming from a charge-transfer event in the hydrogen-bonding motifs responsible for the inter-strand interactions (Schwalb and Temps, 2007, 2008; Saurí et al., 2012). Given the abstruse nature of the photo-processes under study and their intrinsic difficulty, new development in methods and techniques are expected to play a crucial role on disentangling all the different deactivation channels (West et al., 2011, 2012; Krause et al., 2012; West and Moran, 2012; Fingerhut et al., 2013, 2014; Rivalta et al., 2014; Nenov et al., 2014a,b), even though the photophysical and photochemical studies on single nucleobases remain at the cornerstone of the field.

A proper characterization of the monomeric chromophores is therefore essential to understand the photoinduced events occurring in DNA/RNA from a bottom-up approach that can yield definitive answers regarding their role in photostability and photo-damage (Serrano-Andrés and Merchán, 2009). This avenue of research embodies the main efforts carried out by the research community over the last two decades (Kleinermanns et al., 2013; Giussani et al., 2013a; Barbatti et al., 2014; Chen et al., 2014; Mai et al., 2014), and even though plenty of advances have been made there is still no definitive consensus regarding the main deactivation routes present in the nucleobases (Crespo-Hernandez et al., 2004; Hudock et al., 2007; Barbatti et al., 2010; McFarland et al., 2014). Several decay paths have been identified both *in vacuo* (Giussani et al., 2013a) and in solution (Improta and Barone, 2014) and assigned to a number of processes, ranging from a barrierless deactivation through a ring-puckering motion of the bright ππ^*^ state (Merchán and Serrano-Andrés, 2003; Gustavsson et al., 2006; Merchán et al., 2006; Serrano-Andrés et al., 2006, 2008; Conti et al., 2009; Improta and Barone, 2014) to a long-lived signal arising due to a possible crossing with a dark nπ^*^ state (Hare et al., 2007), as well as an even longer-lived pathway widely attributed to the role of triplet states, specially prominent in non-canonical nucleobases with heavy-atom substitutions (Merchán et al., 2005; González-Luque et al., 2010; Martinez-Fernandez et al., 2012, 2014; Pollum et al., 2014).

In this study we turn out attention to water-solvated guanine monophosphate (GMP), one of the canonical DNA/RNA nucleobases. GMP has also been recently proposed to be an interesting compound for nanotechnological applications due to its outstanding capacity for self-assembly (Gupta et al., 2014), specially remarkable in its quadruplex form, which has even been located in DNA/RNA chains (Lam et al., 2013; Murat and Balasubramanian, 2014). Whereas, guanine *in vacuo* has been extensively studied at a high-level multireference *ab initio* level (Serrano-Andrés et al., 2008; Barbatti et al., 2010, 2011) as well as experimentally (Miannay et al., 2010; Chatterley et al., 2014), a lesser degree of scrutiny, mainly at a density functional theory level (Karunakaran et al., 2009; Parac et al., 2010; Santoro et al., 2010; Improta, 2014; Improta and Barone, 2014), has been considered on the effect of the phosphate group and the the solvent on the photoinduced processes occurring in this compound (Crespo-Hernandez et al., 2004). In the present work we propose a theoretical assessment of the deactivation routes embodying the main photophysical and photochemical features of GMP by employing high-level *ab initio* multireference perturbation theory methods coupled with a quantum-mechanical/molecular mechanics (QM/MM) approach, in order to ascertain the role of the environmental perturbations in these type of systems as they remain relatively unknown (Conti et al., 2010; Nachtigallová et al., 2010; Barbatti, 2014). Further knowledge on the environmental effects affecting the photo-processes occurring in the DNA/RNA chromophores upon UV-light irradiation will provide essential information that can be properly translated to water-solvated DNA/RNA systems such as those found in cells.

## Computational details

Sections MM Dynamics and Sampling and QM/MM Calculations describe the computational strategies and methodological details considered in the computations of the MM sampling dynamics and electronic structure QM/MM calculations carried out.

### MM dynamics and sampling

**Figure 1:**
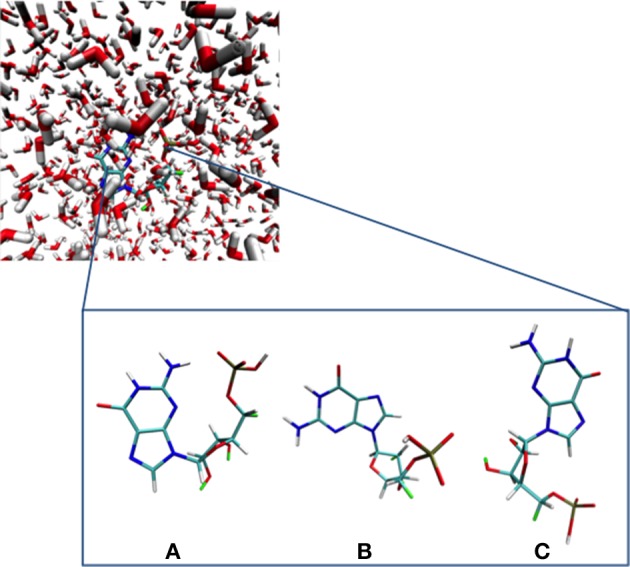
**GMP and its different main conformations along the MM dynamics run obtained through a RMS deviation cluster analysis. (A–C)** depict the most important conformations extracted from the molecular dynamics simulation (see text).

MM simulations were performed for GMP in water to obtain a representative starting geometry to be employed for all subsequent computations and analyses. The MM dynamics calculations were carried out using the Amber-11 suite of programs making use of the Parm99 force field (Case et al., 2005, 2011). Initially, a cubic solvent box comprising 700 water molecules of explicit TIP3P (Jorgensen et al., 1983) with one Na^+^ counterion were considered. The entire system was then heated from 0 to 300 K for 1 ns at constant volume and constant pressure (1 atm), and then finally performing a production run for 100 ns recording the snapshots every 200 fs. To select the initial geometry we performed a cluster analysis based on a Root Mean Square (RMS) coordinate deviation analysis on the guanine moiety over all snapshots recorded along the MM dynamics run within a 2.0 Å difference using the MMTSB toolbox. We obtained three different clusters, denoted a, b, and c in Figure 1, with populations relating to the 93, 6, and 1% of the total number of structures obtained along the dynamics run, respectively. The selected initial structure was therefore chosen as the snapshot with the closest geometrical parameters to the centroid of the average structures obtained in cluster A, being the most representative.

### QM/MM calculations

**Figure 2:**
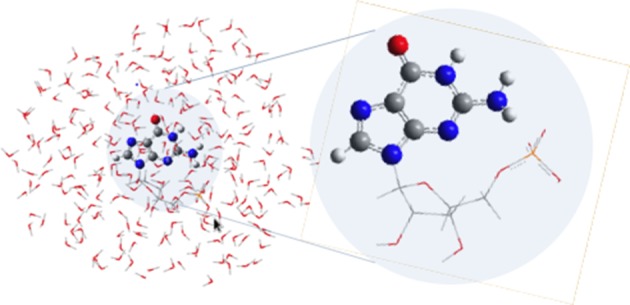
**GMP and the three different QM/MM partitions defined in the present study**. The high layer treating the guanine moiety at the QM level (ball and stick representation), the medium layer encompassing the movable MM water molecules and the phosphate group enclosed within a 10 Å radius distance from GMP, and the low layer containing the remainder of the MM region that is kept frozen throughout the calculations (see text).

QM/MM calculations were performed using the COBRAMM interface developed in our group (Altoé et al., 2007a,b). The cut between the QM and MM regions has been done so that it includes all guanine atoms in the QM region, placing the link atom between the N9 of guanine and the carbon of the sugar ring directly attached to the nucleobase (see Figure 2). The choice of the cut between the QM and MM regions is justified in terms of charge redistribution on the nucleobase and its π-system in order to better reflect the covalent link between guanine and the monophosphate group. A three-layer approach (high, medium, and low) was used throughout (Altoé et al., 2007a): guanine was included in the QM region (high layer, ball, and stick representation in Figure 2), whereas the medium layer comprises the movable MM atoms within a 10 Å radius surrounding the GMP moiety, the remainder of the MM system being kept frozen during all optimization procedures in the low layer. Equilibrium geometries and photoreaction paths (Garavelli, 2006) were determined by using fully unconstrained optimizations and minimum energy path (MEP) computations on the relevant potential energy hypersurfaces by employing the intrinsic reaction coordinate (IRC) and optimization algorithms as implemented in the Gaussian 09 program package (Frisch et al., 2009) interfaced with COBRAMM. CI optimizations were performed with the gradient projection algorithm of Bearpark et al. (1994) as implemented in COBRAMM at the QM/MM level. Further details can be found in Conti et al. (2015).

Energies and gradients in the QM region were computed making use of the complete active space self-consistent field (CASSCF) and complete active space second-order perturbation theory (CASPT2) methods (Andersson et al., 1992; Roca-Sanjuán et al., 2012), as implemented in the MOLPRO-2010 (Werner et al., 2012a,b) and MOLCAS-7 (Aquilante et al., 2010, 2013) suite of programs, respectively. All gradient and non-adiabatic coupling calculations have been performed with the CASSCF implementation of the MOLPRO-2010 code. Subsequent CASPT2 calculations on the key structures obtained along the optimizations and MEPs have been carried out as implemented in the MOLCAS package in order to correct the energy values due to the lack of dynamic correlation present in the CASSCF method. This procedure is referred to as the CASSCF/CASPT2 protocol, and has been successfully employed over the years to tackle a variety of photoinduced phenomena from a theoretical standpoint (Garavelli, 2006; González-Ramírez et al., 2012; Segarra-Martí et al., 2012; Segarra-Martí and Coto, 2014). The active space selected comprised the full π space with the exception of the lowest occupied π orbital plus the three lone-pair orbitals to provide a proper description of the nπ^*^ states, thus making 18 electrons in 13 orbitals. The removal of the lowest occupied π orbital is carried out due to its occupation number being very close to two and given that its removal does not affect the energy values obtained while speeding up the computations. An imaginary level shift of 0.2 a.u. was employed in the perturbation step to avoid intruder states. Two different basis sets were employed throughout: 6-31G^*^ was used in order to map the hypersurfaces at the CASSCF level whereas atomic natural orbital (ANO) type (Pierloot et al., 1995) basis set with the large (ANO-L) primitive set C,N,O(14s9p4d3f)/H(8s4p3d) contracted to O[3s2p1d]/C[3s2p1d]/N[3s2p1d]/H[2s1p] (ANO-L 321/21 hereafter) was employed in order to refine the single-point CASPT2 energies.

## Results and discussion

The results are divided in three different sections: First, the main geometrical parameters of the ground-state Franck-Condon minimum of 9H-guanine both *in vacuo* and in solvated GMP are presented, together with its corresponding vertical spectra, drawing some conclusions on the influence the solvent and phosphate group have on the relative position of the excited states. Next, the different minima and conical intersections describing the photo-process are presented. Finally, a rationale of the photoinduced mechanisms in GMP is drawn in conjunction with previous results and experimental data acquired from the literature, yielding concluding notes on the photophysical and photochemical decay channels featuring in GMP and leading to its intrinsic photostability.

### UV absorption at the Franck-Condon region

**Figure 3:**
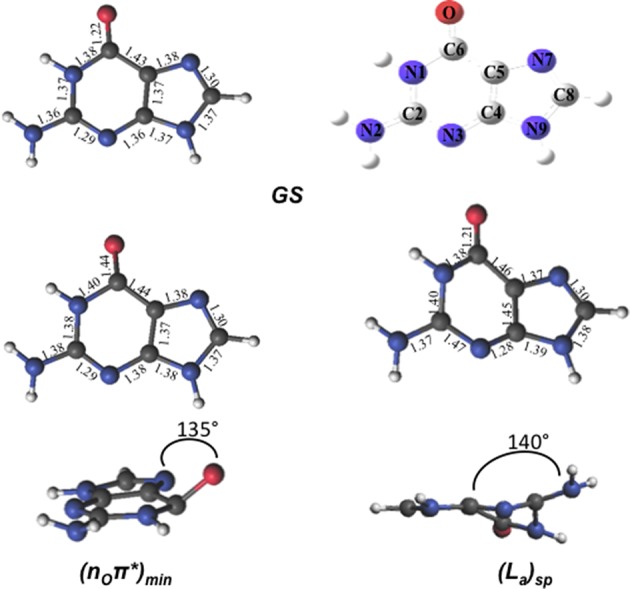
Geometries and main geometrical parameters of the Franck-Condon structure and excited-state stationary points characterized in GMP for its lowest-lying excited states computed at the CASSCF/6-31G^*^ level of theory.

The optimized FC structure of GMP in solution (Figure 3) shows a strong resemblance with the *in vacuo* 9H-Guanine CASSCF/6-31G^*^ structure previously reported in the literature (Serrano-Andrés et al., 2008). Table 1 presents the main geometrical parameters of both structures. As can be seen, bond lengths and dihedral angles are analogous giving rise to a planar structure. Small solvation effects are noticed in the GS structure mainly due to its relatively small dipole moment, providing slight shortenings in the C6-N1 bond distance in the presence of the solvent. A very shallow stationary point (L_a_)_sp_, not present in the gas phase, has been located at the end of the L_a_ MEP featuring a pronounced elongation of the C2-N3 and a shortening of the N3-C4 distances, similar to those featured by the conical intersections between the L_a_ and GS states both *in vacuo* and in solution. The two CIs located between the L_a_ and GS states do show pronounced differences due to the solvent, yielding large elongations in the C2-N3 bond compared to its *in vacuo* counterparts. This is mainly due to the large dipole moment featured by the L_a_ state, which makes it a more influenced state upon solvation. A similar behavior can be seen in the CIs between the L_a_ and L_b_ states, prompting large deviations in the C4-C5, C5-C6 and specially pronounced in the C6-O distance, where water-solvated GMP suffers an elongation of 0.15 Å. The n_O_π^*^ state minimum does not show significant differences highlighting the scarce role polar solvation has on these types of states. The CIs between n_O_π^*^ and the polar ππ^*^ L_a_ and L_b_ states are profoundly influenced by the solvent comparing them to their *in vacuo* counterparts, featuring large differences in the C4-C5 bond distance. These differences are mainly attributed to the polar character of the ππ^*^ L_a_/L_b_ states than to the effects on the n_O_π^*^ state. Overall, it can be concluded that solvation has an important effect on the polar ππ^*^ L_a_/L_b_ states, while being negligible for the n_O_π^*^ state. These states feature excitations that are prominently placed on the six-member ring of guanine featuring noticeable changes in the structure upon solvation, whereas the five-member ring remains relatively unchanged. A possible cause for this effect could lie on the presence of the phosphate group, which is tied to the five-ring member and could be shielding that molecular moiety from the surrounding water molecules thus mitigating its exposure as compared to the six-member ring and justifying why the latter suffers such pronounced changes upon solvation.

**Table 1:** **Bond distances (in Å) characterizing the key structures involved in the photoinduced events of GMP upon UV-light irradiation**.

	**N1-C2**	**C2-N2**	**C2-N3**	**N3-C4**	**C4-C5**	**C5-C6**	**C6-O**	**C6-N1**	**C5-N7**	**N7-C8**	**C8-N9**	**N9-C4**
GS *in vacuo*^a^	1.37	1.38	1.31	1.36	1.39	1.44	1.21	1.41	1.38	1.30	1.38	1.37
GS in solution ^b^	1.37	1.36	1.29	1.36	1.37	1.43	1.20	1.38	1.38	1.30	1.37	1.37
(L_a_)_sp_ in solution^b^	1.40	1.37	1.47	1.28	1.45	1.46	1.21	1.38	1.37	1.30	1.38	1.39
(L_a_/GS)_CI-1_ *in vacuo*^a^	1.41	1.40	1.45	1.29	1.45	1.47	1.20	1.41	1.39	1.29	1.39	1.37
(L_a_/GS)_CI-1_ in solution^b^	1.37	1.38	1.50	1.24	1.48	1.47	1.23	1.37	1.37	1.30	1.38	1.42
(L_a_/GS)_CI-2_ *in vacuo*^a^	1.41	1.40	1.38	1.28	1.45	1.35	1.34	1.36	1.39	1.29	1.38	1.38
(L_a_/GS)_CI-2_ in solution^b^	1.42	1.36	1.49	1.27	1.48	1.48	1.21	1.39	1.38	1.30	1.39	1.39
(L_b_/L_a_)_CI_ *in vacuo*^a^	1.38	1.40	1.32	1.31	1.45	1.43	1.24	1.36	1.35	1.44	1.42	1.40
(L_b_/L_a_)_CI-1_ in solution^b^	1.39	1.37	1.28	1.37	1.38	1.38	1.39	1.39	1.38	1.30	1.36	1.38
(L_b_/L_a_)_CI-2_ in solution^b^	1.46	1.35	1.37	1.21	1.59	1.54	1.18	1.30	1.30	1.47	1.41	1.39
(n_O_)_min_ *in vacuo*^a^	1.38	1.38	1.28	1.37	1.36	1.44	1.38	1.41	1.36	1.30	1.37	1.36
(n_O_)_min_ in solution^b^	1.38	1.38	1.29	1.38	1.36	1.44	1.41	1.40	1.38	1.30	1.37	1.80
(n_O_/L_a_)_CI_ *in vacuo*^a^	1.41	1.40	1.38	1.28	1.45	1.35	1.34	1.36	1.39	1.29	1.38	1.38
(n_O_/L_a_)_CI_ in solution^b^	1.39	1.37	1.28	1.37	1.38	1.38	1.39	1.39	1.38	1.30	1.36	1.38
(n_O_/L_b_)_CI_ in solution^b^	1.40	1.35	1.30	1.36	1.38	1.42	1.24	1.36	1.37	1.30	1.37	1.37

a Values obtained at the CASSCF/6-31G^*^ level of theory by Serrano-Andrés et al. (2008).

b Values computed in the present study.

Table 2 contains the vertical excitation energies at the CASPT2 level computed in the present study, together with several other computations and experimental values reported in the literature. We have used Platt's nomenclature (Platt, 1949), where L_a_ represents the ππ^*^ excited state characterized by the largest contribution of the configuration HOMO (H) → LUMO (L) to the CASSCF wave function, whereas L_b_ depicts the ππ^*^ excited state with a predominance of H → L+1 and H-1 → L configurations. By inspection of Table 2, it can be readily seen that there is a qualitative difference in the relative order of the lowest-lying excited states *in vacuo* and in solution: both cases feature the π π^*^ L_a_ state as the energetically lowest-lying excited state, at 4.93 and 4.77 eV respectively, but while *in vacuo* S_2_ has n_O_π^*^ nature (5.54 eV, involving the oxygen lone pair of the purinic base), in solution S_2_ isthe ππ^*^ L_b_ state, placed at 5.44 eV. This effect has been widely reported in the literature being related to a bathochromic effect (Nakayama et al., 2013), with the n_O_π^*^ featuring a dipole moment of 5.69 D (3.51 D *in vacuo*) being less stabilized than the ππ^*^ L_b_ state (7.96 D, 4.92 D *in vacuo*) as it can be also noticed by looking at their change in dipole moment. The solvent therefore affects the absorption spectra prominently at the FC region by blue-shifting the n_O_π^*^ state by 0.23 and red-shifting the ππ^*^ L_b_ by 0.33 eV with respect to the gas-phase, a change comparable to those previously reported in other QM/MM studies in solution (Parac et al., 2010; Nakayama et al., 2013). It is worth noting that the oscillator strengths associated to the transitions to the L_a_ and L_b_ states are also affected by the effect of the solvent. Whereas, L_a_ features as the brightest lowest-lying excited state in the gas phase and the L_b_ state presents a slightly smaller oscillator strength (0.158 and 0.145 respectively), in solvent L_a_ and L_b_ present an inverse order in the values for the oscillator strength associated to their transitions (0.09 and 0.17 respectively), being in agreement with the experimental data reported by femtosecond broad-band transient spectroscopy (Karunakaran et al., 2009).

**Table 2:** **Vertical absorption energies (ΔE, in eV), oscillator strengths (*f*) and dipole moments (μ, in Debye) computed at the FC region, together with previous theoretical and experimental data**.

**States**	**ΔE**	***f***	**μ**
**9H-GUANINE *IN VACUO*^a^**			
GS	0		5.81
ππ^*^ L_a_	4.93	0.158	5.23
n_O_π^*^	5.54	0.002	3.51
π π^*^ L_b_	5.77	0.145	4.92
GMP IN SOLVENT (6-31G*)^b^			
GS	0		8.58
ππ^*^ L_a_	4.77	0.09	8.97
n_O_π^*^	5.77	0.00	5.69
ππ^*^ L_b_	5.44	0.17	7.96
**GMP IN SOLVENT (ANO-L 321/21)^b^**			
GS	0		8.88
ππ^*^ L_a_	4.50	0.17	9.62
n_O_π^*^	5.71	0.00	5.64
ππ^*^ L_b_	5.10	0.20	8.31
**EXPERIMENT^c^**			
ππ^*^ L_a_	4.50	0.094	
n_O_π^*^			
ππ^*^ L_b_	4.96	0.167	
9Me-GUANINE^*^5H2O (TDDFT/PCM)^c^			
ππ^*^ L_a_	4.68	0.16	
n_O_π^*^	5.77		
ππ^*^ L_b_	5.18	0.34	
**9H-GUANINE (TDDFT/TZVP)^d^**			
ππ^*^ L_a_	4.93	0.158	
n_O_π^*^	5.36	0.106	
ππ^*^ L_b_	5.28	0.117	
**9H-GUANINE (DFT/MRCI/TZVP)^d^**			
ππ^*^ L_a_	4.83	0.22	
n_O_π^*^	5.32	0.001	
ππ^*^ L_b_	5.08	0.368	
⊥tomrule

a Values obtained at the CASSCF/6-31G^*^ level of theory by Serrano-Andrés et al. (2008).

b Results obtained in the present study.

c Results obtained by Karunakaran et al. (2009).

d Results obtained by Parac et al. (2010).

The effect of the basis set on the excitation energies has also been studied by employing both 6-31G^*^ and ANO-L 321/21 basis sets. The 6-31G^*^ CASPT2 computations report values of 4.77, 5.44, and 5.77 eV for the L_a_, L_b_, and n_O_π^*^ transitions, respectively, whereas the ANO-L yields absorption energy values of 4.50, 5.10, and 5.71 eV for the L_a_, L_b_, and n_O_π^*^ states respectively, consistent with those reported experimentally (Karunakaran et al., 2009), which place the absorption maximum of L_a_ at 4.50 eV. Table 2 also reports several theoretical approaches found in the literature employing different methods to simulate solvated GMP. As can be seen, more sophisticated DFT/MRCI methods agree qualitatively with the values here reported at the CASPT2 level of theory, yielding values within 0.33 and 0.02 for the L_a_ and L_b_ states respectively with respect to the CASPT2/ANO-L values here obtained, and a slight energetic deviation is also found when comparing to the values computed at a TDDFT level. This small difference present in TDDFT results employing a polarization continuum model (PCM) (see Table 2) could also be due to the fact that (Karunakaran et al., 2009) used a methylated guanine to model the effect of the phosphate group whereas here the phosphate group is explicitly included even if just at the MM level. On overall we can conclude that the absorption values reported here at the CASPT2/ANO-L level are consistent with the experimental data and with the highest-level theoretical estimates present in the literature, thus highlighting the capabilities of the CASSCF/CASPT2 protocol to treat excited states in a balanced manner (Roos et al., 1996) and its usage for mapping photochemical reaction paths (Garavelli, 2006).

### Excited-state evolution

The excited-state evolution is here tracked by means of static approaches through excited-state optimizations and the characterization of the conical intersections representing the crossings among the energetically lowest-lying excited states, ultimate protagonists in the deactivation photo-process. Additionally, the MEPs connecting the initially accessed states and subsequent photoinduced events have been mapped making use of the IRC technique.

**Figure 4:**
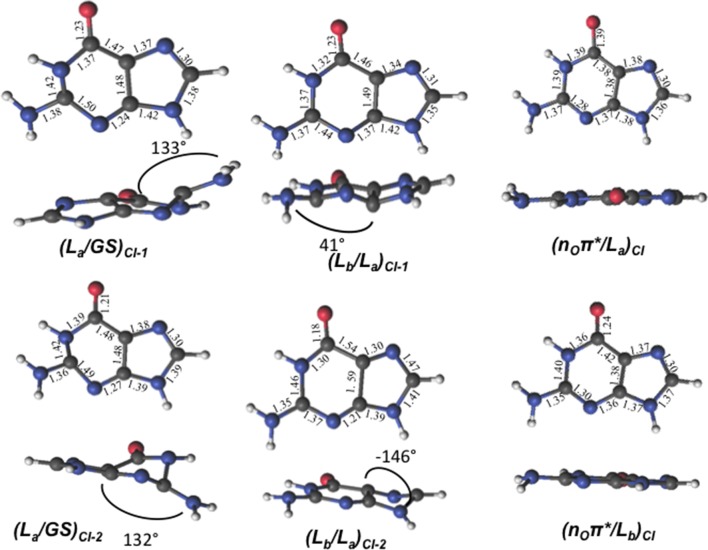
GMP geometries and main geometrical parameters of the characterized conical intersections between the lowest-lying excited states computed at the CASSCF/6-31G* level of theory.

**Figure 5:**
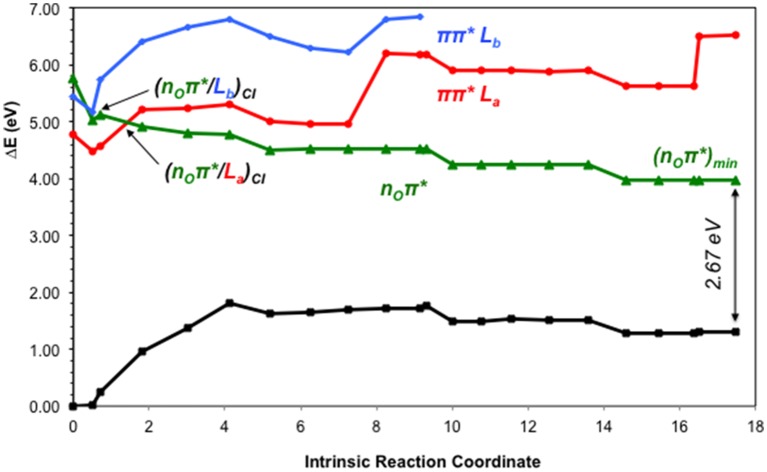
CASPT2 energies of the ground (GS) and lowest-lying singlet excited states (ππ^*^ L_a_, ππ^*^ L_b_ and n_O_π^*^) of the GMP from the FC geometry and along the ππ^*^ L_a_ CASSCF MEP.

The lowest-lying excited state is the ππ^*^ L_a_ state, which is expected to be the main spectroscopic state due to its lowest-lying position and relatively large oscillator strength. This state is generally assumed to present an easily accessible CI along its relaxation pathway toward the ground-state characterized by a ring-puckering motion (see Figure 4) widely featured in the DNA/RNA nucleobases (Giussani et al., 2013a) *in vacuo*. This profile is slightly altered in the presence of polar environments as it has been previously reported for other purine nucleobases (Conti et al., 2010), where a shallow stationary point (L_a_)_sp_ arises along the MEP close to the CI with the ground state being placed at 1.15 eV vertically and adiabatically at 3.30 eV from the ground state (see Figure 5). Two different CIs have been characterized in the vicinity of this stationary point, one optimized directly in solvent corresponding to the minimum energy conical intersection (MECI) also reported by Serrano-Andrés et al. (2008), and another relating to the CI found in the same study along the MEP *in vacuo*, which we have tentatively named (L_a_/GS)_CI-1_ and (L_a_/GS)_CI-2_ in the present study, respectively. Both CIs as well as the (L_a_)_sp_ present ring-puckering structures in the A6 cycle yielding slight bond-length alterations (C2-N3, N1-C2, N3-C4, and C4-C5) compared to the FC structure and featuring prominent N1-N-C2-N3 dihedral angle distortions at 133°, 132°, and 140° for the (L_a_/GS)_CI-1_, (L_a_/GS)_CI-2_, and (L_a_)_sp_ structures, respectively. These distortions are quantitatively different to those reported *in vacuo*, stressing out the importance of the solvent where the (L_a_/GS)_CI-1_ presents a ~143° dihedral angle as compared to its 133° solvated counterpart, together with slightly pronounced bond-length differences as can be seen in Table 1. Both CIs here characterized present very similar structures (see Figure 4) and charge distributions very close to those found in the gas phase, presenting an inversion in their energetic order in solution being (L_a_/GS)_CI-1_ the energetically lowest-lying adiabatically at 3.31 eV, (L_a_/GS)_CI-2_ being placed 0.3 eV higher in energy and 3.60 eV adiabatically from the FC region.

Two different CIs connecting the ππ^*^ L_a_ and ππ^*^ L_b_ states have also been located. Direct CI optimization leads to (L_b_/L_a_)_CI-2_, its geometry presenting a deformation near the N9-C8-N7 angle with respect to the ground state. The bond lengths suffer large distortions, observing a shortening of the N1-C6, C5-N7, C4-N3, N3-C2, C6-O, and C2-N bonds, and an increase in the length of the remaining bonds with respect to the FC region (see Figure 4). This CI is placed at 7.62 eV vertically from the FC region, which is higher in energy than both ππ^*^ L_a_ and ππ^*^ L_b_ vertical excitation energies, placed at 4.77 and 5.44 respectively, thus hinting toward a sloped CI profile preventing their non-adiabatic interaction. A second CI, (L_b_/L_a_)_CI-1_, obtained along the L_b_ MEP computed within a 4in4 active space to avoid excessive wave function mixing, has been located being placed ~5 eV adiabatically from the FC region. This CI presents geometrical similarities with its *in vacuo* counterpart and provide an accessible channel to funnel the initially populated L_b_state down to L_a_. Further attempts to optimize the L_b_ state toward a possible minimum have been fruitless due to the large wave function mixing and root flipping problems experienced along the optimization procedure, preventing us to obtain further information on this specific state.

**Figure 6:**
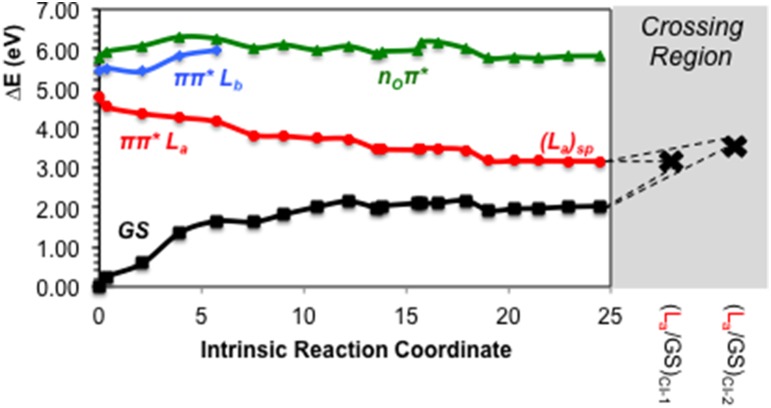
CASPT2 energies of the ground (GS) and lowest-lying singlet excited states (ππ^*^ L_a_, π π^*^ L_b_ and n_O_π^*^) of the GMP from the FC geometry and along the n_O_π^*^ CASSCF MEP.

As previously stated, n_O_π^*^ represents the third energetically wise excited state for the GMP in solvent. The optimized n_O_π^*^ minimum found in solvent is prominently characterized by an elongation of the C6-O bond distance with respect to the ground state. Table 1 shows the main differences in bond lengths, where the C6-O bond is elongated in this minimum from 1.22 Å at the FC region to 1.41 Å. The minimum of this excited state in solvent is very similar to the one reported *in vacuo* (Serrano-Andrés et al., 2008), as would be expected given the small effects provided by the solvent on nπ^*^ excited states. Energetically it is placed 3.97 eV adiabatically and 2.67 eV vertically with respect to the ground state (see Figure 6), which constitutes a stabilization of 1.8 eV from its initial value at the FC region.

We have also located the CI between the states n_O_π^*^ and π π^*^ L_a_, (n_O_/L_a_)_CI_, its geometry presenting a planar structure like the ground state, but showing an elongation of the C6-O bond and a shortening of the C6-C5 bond. We observe similarities between this geometry and that of the CI optimized *in vacuo*, as they both present the same kind of geometrical distortions given the small solvent effects in the n_O_π^*^ state, yielding a relative energy of 4.90 eV with respect to the FC region. The CI connecting the n_O_π^*^ and π π^*^ L_b_ states, (n_O_/L_b_)_CI_, has also been located, featuring a planar structure and differences in the bond lengths in the six-member ring, in particular in the differences in the N1-C2, N1-C6 bonds.

The differences observed in the geometries of the excited state minima and of the different CIs characterized both *in vacuo* and in solvent highlight the importance of the solvent that is considered explicit in our calculations, together with the explicit presence of the ribose and phosphate groups on GMP, providing more accurate estimates to relate to the photoinduced events in the cellular system.

### Photophysics and photochemistry of GMP: elucidating its intrinsic photostability

**Figure 7:**
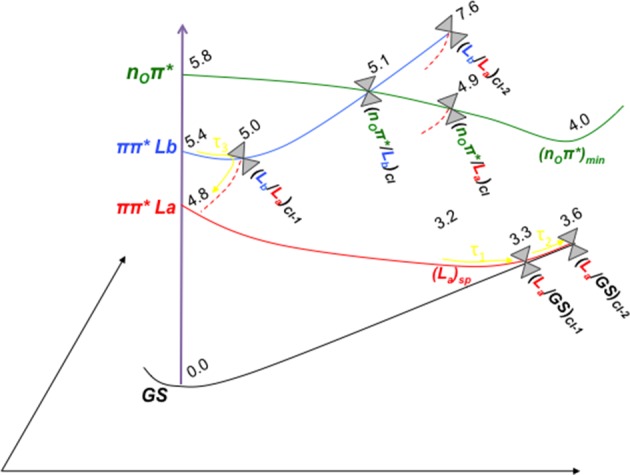
**Scheme of the photoinduced processes occurring in solvated GMP upon UV-light irradiation**. The energetic values (in eV) and the different crossing points and minima depicted refer to the CASPT2 computations carried out in the present study. The associated time constants marked in yellow have been taken tentatively from the ultrafast pump-probe transient absorption experiments reported by Karunakaran et al. (2009). Three different decay channels have been assigned to the experimental evidence: τ_1_ refers to the ultrafast decay path from an initially accessed π π^*^ L_a_ excitation to the ring-puckering CI with the GS, τ_2_ to the initial population of the π π^*^ L_b_ state and subsequent non-adiabatic population transfer to the L_a_ state finalizing in the CI between L_a_ and GS, and τ_3_ that is tentatively assigned mainly to deactivation processes along the L_b_ state (See text for details).

Figure 7 features a scheme with the tentative photo-processes occurring upon UV irradiation in GMP based on our present computations and the theoretical and experimental data available in the literature. Experimentally, time-resolved transient absorption in solution by Karunakaran et al. (2009) suggest either bi- or triexponential decay depending on the spectral region measured, providing τ_1_ = 0.25 ps, τ_2_ = 1.0 ps, and τ_3_ = 2.5 ps for the 270–400 nm region in the non-protonated GMP at neutral pH. (Karunakaran et al., 2009) justify these ultrafast signals in terms of TDDFT calculations due to ring-puckering CIs between the π π^*^ L_a_ and GS states, in accordance to what was previously proposed *in vacuo* by Serrano-Andrés et al. (2008) on the basis of CASSCF/CASPT2 computations. Molecular dynamics simulations by Serrano-Andrés et al. (2008) proposed that the ultrafast decay from the π π^*^ L_a_ state could occur already within the first 100 fs, an assumption that has been challenged by Barbatti et al. (2011), pushing the timescale toward the 500 fs mark. (Lan et al., 2009), on the other hand, support a biexponential decay based on their MD simulations where they obtain a faster ~190 fs and a slower ~400 fs decays through two different CIs between the π π^*^ L_a_ and GS states, thus highlighting the complex process under study and the difficulty to simulate it coherently (Mai et al., 2014). The most recent experimental data to our knowledge is based on the photoelectron spectroscopy of GMP both *in vacuo* and in solution (Chatterley et al., 2014), yielding a biexponential decay of τ_1_ = 50 fs and τ_2_ = 600 fs *in vacuo* and τ_1_ = 120 fs and τ_2_ = 680 fs in solution, which reveals a striking similarity among the two different set of values as they are within experimental error and therefore provide evidence of the negligible role played by the solvent in the photoinduced decay paths present in this system. This biexponential decay is obtained through a 4.66 eV pump, which also suggests the possibility of only probing those kinetic processes undergone after direct π π^*^ L_a_ population, whereas the time-resolved transient data by Karunakaran et al. (2009) yields a third exponent related to a slower timescale possibly arisen through an initial population of the π π^*^ L_b_ state.

Our present results point toward an ultrafast deactivation along the L_a_ MEP characterized by a ring-puckering motion centered in the C2 atom, which brings the excited-state population to interact non-adiabatically with the ground state, funneling down the population that is further relaxed to the FC region. This kind of process has been widely attributed to the DNA/RNA nucleobases and is permanently linked with their intrinsic photostability (Serrano-Andrés and Merchán, 2009) and therefore extends to the photostability of the genomic material. Our calculations in the presence of the solvent and the sugar and phosphate groups reveal a very shallow stationary point at the end of the MEP right before reaching the intersection seam (see Figure 5), which has also been previously documented in other nucleobases at a TDDFT level of theory to be related to a transition state with an imaginary reaction coordinate that drives the system toward the ring-puckering CI (Picconi et al., 2011). This stationary point is explained in terms of the planarity of the π π^*^ potential energy hypersurfaces of all nucleobases (Giussani et al., 2013a; Improta and Barone, 2014), and is expected to be overcome in order to reach the CIs with the ground state in an ultrafast manner. In contrast, we have shown recently (Conti et al., 2010, 2015) that the corresponding internal conversion path in adenine is controlled by a larger barrier, which may explain its longer lifetimes. Two different CIs have been located between the π π^*^ L_a_ and GS states, namely (L_a_/GS)_CI-1_ and (L_a_/GS)_CI-2_. These two CIs are analogous to those found *in vacuo* by Serrano-Andrés et al. (2008) but present an energetic inversion due to the embedding effect of the solvent. Both are expected to belong to the same intersection seam given their similar structures, and both are considered to be accessible from the L_a_ stationary point previously characterized. (L_a_/GS)_CI-1_, being the lowest-lying energy wise is expected to embody the fastest decay route, relating to the τ_1_ = 0.25 ps recorded through time-resolved transient and τ_1_ = 120 fs in time-resolved photoelectron spectroscopy experiments. This assignment is analogous to that previously reported for 9H-Guanine *in vacuo*, and supports recent experiments reporting analogous excited-state decays for GMP in gas-phase and in solution (Chatterley et al., 2014). The second conical intersection with the ground state, (L_a_/GS)_CI-2_, might be responsible for the second exponential measured at τ_2_ = 1.0 ps and τ_2_ = 680 fs in time-resolved transient and photoelectron spectroscopy, respectively, given its higher-lying energetic position and a relatively small barrier of ~0.3 eV to be overcome in order to access it once reaching the intersection seam. Nevertheless, the existence of another CI between the L_a_ and GS states not characterized in the present contribution could better explain that second lifetime, which will be related to the L_a_ surface as it has been recently reported experimentally (Chatterley et al., 2014). These assignments are in line with the MD studies by Lan et al. (2009), reporting two different decay routes with their associated timescales to two distinct CIs between the π π^*^ L_a_ and the ground state, and are in line with other theoretical CASPT2 and TDDFT studies that point toward the L_a_ state as the responsible for both ultrafast decay timescales.

The π π^*^ L_b_ state is predicted to be involved in the photoinduced events being related possibly only to the longest-lived τ_3_. This is explained in terms of an initial population of the L_b_ state, which presents a sizable oscillator strength, and subsequent funneling of the population toward the L_a_ state mediated by the (L_b_/L_a_)_CI-1_, placed at ~5 eV along the L_b_ relaxation pathway. The second CI characterized between L_b_ and L_a_ states, (L_b_/L_a_)_CI-2_, is expected to be irrelevant to the photo-process due to its high-lying energetic position preventing its accessibility. The lone-pair excited state, n_O_π^*^, is blue-shifted in solution as has been already mentioned above. Its elevated vertical absorption energy prevents it to be one of the main spectroscopic states, yet its close-lying position to the absorbing π π^*^ L_b_ state facilitates a possible population of this state and subsequent relaxation toward its minimum, (n_O_π^*^)_min_. This minimum is placed at 3.97 eV adiabatically and 2.67 eV vertically from the GS, and could be partly responsible of the longest-lived signal reported experimentally at τ_3_ = 2.5 ps or at τ_3_ = 167 ps at low pH, given that such nπ^*^-mediated processes have been already characterized experimentally to be close to the ~100 ps timescale on pyrimidines both *in vacuo* and in solution (Hare et al., 2007).

The present study elucidates the photoinduced events in GMP in terms of an ultrafast decay along the main spectroscopic and initially accessed π π^*^ L_a_ state characterized by a ring-puckering motion, which would cover the experimental timescales τ_1_ and τ_2_ through different CIs with the ground state, whereas the longest-lived component would be attributed to the decay routes mediated through the π π^*^ L_b_ and, to a minor extent, to the n_O_π^*^ state.

## Conclusions

The present study encompasses a photophysical and photochemical appraisal of water-solvated GMP by means of theoretical multireference perturbation theory QM/MM techniques. An initial MD simulation has been carried out in order to characterize the geometrical parameters of the FC region. The vertical excitation energies have been computed and compared to recent data found in the literature and to the results obtained *in vacuo*, highlighting the importance of the environment yielding qualitative differences for the π π^*^ L_a_ and π π^*^ L_b_ states being red-shifted and for the n_O_π^*^ state being blue-shifted as compared to their gas-phase counterpart. The π π^*^ L_a_ state is predicted to be the main spectroscopic state driving the ultrafast deactivation processes occurring in GMP upon UV-light irradiation based on a ring-puckering motion that enhances its non-adiabatic interaction with the ground state in a radiationless manner. A shallow stationary point toward the end of the π π^*^ L_a_ MEP has been characterized, together with two different CIs with the ground state that help rationalize the two fastest decay times measured experimentally. Upon initial L_b_ absorption, two CIs between the π π^*^ L_b_ and L_a_ states have also been located, one of them along the L_b_ decay path pointing toward a population funneling down to the L_a_ state and another being too high in energy to contribute prominently in the photo-process. The CIs connecting the n_O_π^*^ state and the π π^*^ L_b_ and L_a_ states have also been characterized along its relaxation route, yielding a minimum in the n_O_π^*^ state expected to emit vertically at ~2.7 eV. Both π π^*^ L_b_ and n_O_π^*^ are suggested to contribute to the longest-lived experimental timescale, the latter providing a lesser contribution given the relatively fast kinetic timescale and the long-lived timescales expected in nπ^*^ fluorescent states. Upcoming QM/MM dynamics simulations are expected to shed some more light on the photoinduced events occurring in water-solvated GMP and its specific decay timescales in order to provide a more specific molecular counterpart to the experiment and better explain the photochemical and photophysical processes resulting in the intrinsic stability of the genomic material.

### Conflict of interest statement

The authors declare that the research was conducted in the absence of any commercial or financial relationships that could be construed as a potential conflict of interest.
