# Supplementary material for: Deciphering the photochemical mechanisms describing the UV-induced processes occurring in solvated guanine monophosphate
Source: Front Chem. 2015 Apr 20;3:29. doi: 10.3389/fchem.2015.00029 (PMC4403598; doi:10.3389/fchem.2015.00029)
Supplement: Supplementary file 1 [file DataSheet1.DOCX]

***Supplementary Material***

*(Total of 5 pages)*

for

*Deciphering the photochemical mechanisms describing the UV-induced processes occurring in solvated guanine monophosphate*

Salvatore F. Altavilla^1^, Javier Segarra-Martí^1,a)^ , Artur Nenov^1^, Irene Conti^1^, Ivan Rivalta^2^, and Marco Garavelli^1,2,b)^

^1^ Dipartimento di Chimica “G. Ciamician, Università di Bologna, Via Selmi 2, IT-40126 Bologna, Italy.

^2^ Université de Lyon, CNRS, Institut de Chimie de Lyon, École Normale Supérieure de Lyon, 46 Allée d’Italie, F-69364 Lyon Cedex 07, France.

1. [Javier.Segarra@unibo.it](mailto:Javier.Segarra@uv.es)
2. [marco.garavelli@unibo.it](mailto:pedro.brana-coto@physik.uni-erlangen.de)

**Cartesian coordinates (x, y and z in Å) of all the structures (just QM part) discussed in the paper.**

**GS**

**x y z**

O 19.057404 13.774774 15.475440

N 15.928063 15.879140 18.067523

N 18.037686 15.151293 18.030972

N 17.030106 14.257779 14.621401

N 14.980602 14.592381 13.594251

N 15.246615 15.238369 15.796241

C 17.121159 15.740257 18.735567

C 17.433642 14.898767 16.820905

C 17.959007 14.276730 15.640705

C 15.736960 14.708820 14.722035

C 16.132277 15.333817 16.824119

H 17.248020 16.095761 19.735572

H 17.379643 13.933484 13.733828

H 15.324200 14.021084 12.848169

H 13.992068 14.469706 13.742201

H 15.055080 16.205422 18.451586

**(L_a_)_sp_**

**x y z**

N 15.898564 16.121369 18.060407

C 16.996363 15.691424 18.788305

H 17.145960 16.009006 19.800548

N 17.797343 14.899349 18.145826

C 17.222654 14.715188 16.910296

C 17.968863 14.397371 15.693377

O 19.069609 13.890116 15.671967

N 17.260555 14.730485 14.552915

H 17.667837 14.449918 13.676401

C 15.893180 14.597208 14.832924

N 15.091495 14.198930 13.810164

H 15.429867 13.455398 13.234245

H 14.101887 14.157802 13.975821

N 15.357867 15.637385 15.718040

C 16.005041 15.497287 16.821141

H 15.042239 16.472269 18.459959

**(L_a_/GS)_CI-1_**

**x y z**

N 15.950909 16.305139 18.091417

C 16.991843 15.791774 18.836343

H 17.144730 16.105984 19.849132

N 17.757791 14.943010 18.222107

C 17.223193 14.802642 16.967285

C 18.024094 14.519416 15.766755

O 19.123867 13.960282 15.815557

N 17.391732 14.902635 14.612323

H 17.789055 14.578139 13.749355

C 16.021277 14.709489 14.912013

N 15.221471 14.273098 13.874335

H 15.595977 13.488368 13.379490

H 14.237879 14.199700 14.040795

N 15.486757 15.848798 15.728700

C 16.028342 15.672972 16.826016

H 15.094847 16.675279 18.473803

**(L_a_/GS)_CI-2_**

**x y z**

N 15.664271 15.969737 17.855122

C 16.936728 15.999634 18.406455

H 17.092442 16.353591 19.404434

N 17.885540 15.564763 17.634464

C 17.277304 15.272921 16.434671

C 17.734198 14.195476 15.522252

O 18.837501 13.701983 15.537211

N 16.724901 13.825672 14.639406

H 17.054660 13.387543 13.793716

C 15.991201 15.033678 14.463245

N 15.467518 15.224978 13.221743

H 15.953506 14.976182 12.388755

H 14.695138 15.817957 13.043126

N 15.042047 15.251407 15.592801

C 15.831520 15.556354 16.539699

H 14.808071 16.296828 18.274642

**(L_b_/L_a_)_CI_**

**x y z**

N 15.898352 15.949700 18.043461

C 17.102551 15.847250 18.646564

H 17.297056 16.299634 19.598737

N 18.025249 15.196212 17.981169

C 17.425765 14.798820 16.845389

C 17.980819 14.236007 15.622769

O 19.112164 13.757881 15.493919

N 17.081742 14.216677 14.651952

H 17.434487 13.853200 13.764226

C 15.762392 14.595975 14.688189

N 15.020093 14.632002 13.536398

H 15.371374 14.092276 12.769977

H 14.038245 14.445658 13.680585

N 15.298120 15.463424 15.738730

C 15.967770 15.128477 16.881179

H 15.037495 16.272305 18.456880

**(n_O_)_min_**

**x y z**

N 15.909906 15.887393 18.172244

C 17.112799 15.845582 18.818469

H 17.224039 16.193472 19.823841

N 18.070559 15.352359 18.087298

C 17.470332 15.062221 16.883064

C 18.058516 14.693822 15.620908

O 18.989661 13.630277 15.603194

N 17.104438 14.507677 14.615102

H 17.456667 14.400669 13.682110

C 15.765114 14.778457 14.789353

N 14.998086 14.619948 13.661609

H 15.354523 14.015475 12.948674

H 14.022900 14.443682 13.840920

N 15.237890 15.183806 15.890858

C 16.141972 15.368133 16.915435

H 15.034624 16.230828 18.535560

**(n_O_/L_a_)_CI_**

**x y z**

N 15.921259 15.888625 18.078984

C 17.115561 15.741524 18.735815

H 17.251354 16.092521 19.735062

N 18.041943 15.148173 18.032994

C 17.450426 14.891642 16.821311

C 17.917892 14.309792 15.673099

O 19.089236 13.749929 15.461219

N 17.032838 14.242267 14.596084

H 17.386809 13.963758 13.700538

C 15.741968 14.700506 14.713597

N 14.975066 14.592878 13.589496

H 15.317946 14.023531 12.841813

H 13.989423 14.458986 13.741997

N 15.248943 15.243094 15.803852

C 16.107573 15.343327 16.823615

H 15.045982 16.211832 18.460417

**(n_O_/L_b_)_CI_**

**x y z**

N 15.927910 15.879702 18.069056

C 17.119857 15.740516 18.735516

H 17.248723 16.092443 19.734768

N 18.038271 15.150500 18.030109

C 17.437844 14.896161 16.819692

C 17.947082 14.283870 15.648240

O 19.065370 13.768405 15.473627

N 17.029662 14.256179 14.616784

H 17.373939 13.935448 13.729378

C 15.737879 14.707720 14.720611

N 14.980205 14.592088 13.593898

H 15.322449 14.021780 12.845522

H 13.991121 14.468886 13.739892

N 15.246631 15.239106 15.797726

C 16.128540 15.335036 16.822757

H 15.054414 16.205648 18.452237
